# Supplementary material for: Electrodeposition of Nanoparticles and Continuous Film of CdSe on n-Si (100)
Source: Nanomaterials (Basel). 2019 Oct 22;9(10):1504. doi: 10.3390/nano9101504 (PMC6835584; doi:10.3390/nano9101504)
Supplement: Supplementary file 1 [file nanomaterials-09-01504-s001.pdf]

nanomaterials

MDPI

SUPPORTING INFORMATION

Electrodeposition of nanoparticles and continuous film of CdSe on n-Si (100)

Walter Giurlani [1], Vincenzo Dell'Aquila [1], Martina Vizza [1], Nicola Calisi [1], Alessandro Lavacchi [2], Alessia Irrera [3], Maria Josè Lo Faro[4], Antonio Alessio Leonardi[3,4], Dario Morganti[3,4] and Massimo Innocenti [1,*]

[1] Dipartimento di Chimica, Università degli Studi di Firenze, via della Lastruccia 3, 50019 Sesto Fiorentino, Italy

[2] CNR-ICCOM, Istituto di Chimica dei Composti OrganoMetallici, via Madonna del Piano 10, 50019 Sesto Fiorentino (FI), Italy;

[3] CNR-IPCF, Istituto per i Processi Chimico-Fisici, V.le F. Stagno D'Alcontres 37, 98158 Messina, Italy;

[4] Dipartimento di Fisica ed Astronomia, Università di Catania, Via Santa Sofia 64, 95123 Catania, Italy;

* Correspondence: m.innocenti@unifi.it;

We report the SEM images of sample A (Cd on n-Si, Figure S1) and sample B (Se on n-Si, Figure S2) at same high magnification of Sample C and B.

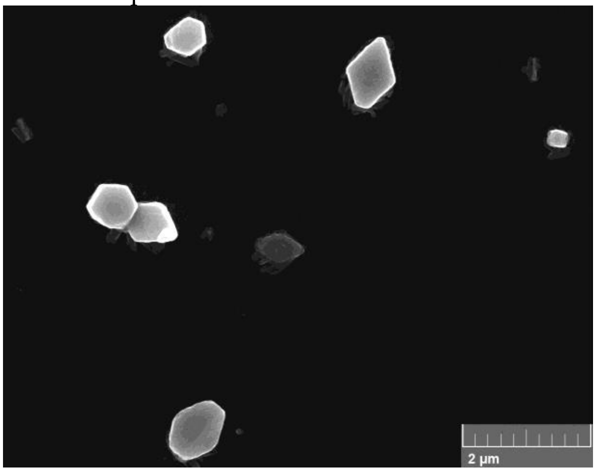

Figure S1. SEM analysis of sample A (of 30 nmol of Cd deposited on n-Si) at high magnification.

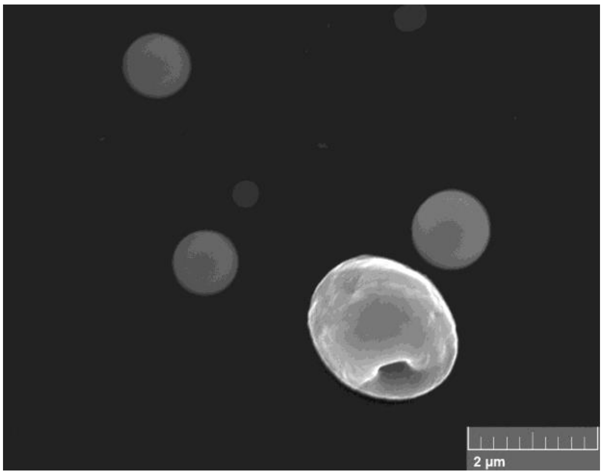

Figure S2. SEM analysis of sample B (30 nmol of Se deposited on n-Si) at high magnification.

In Figure S3 we report the electrochemical behavior of Cd^{2+} on Se/n-Si (Figure S3a) and Se(IV) on Cd/n-Si (Figure S3b). The absence of the anodic peak of cadmium suggest the formation of a compound.

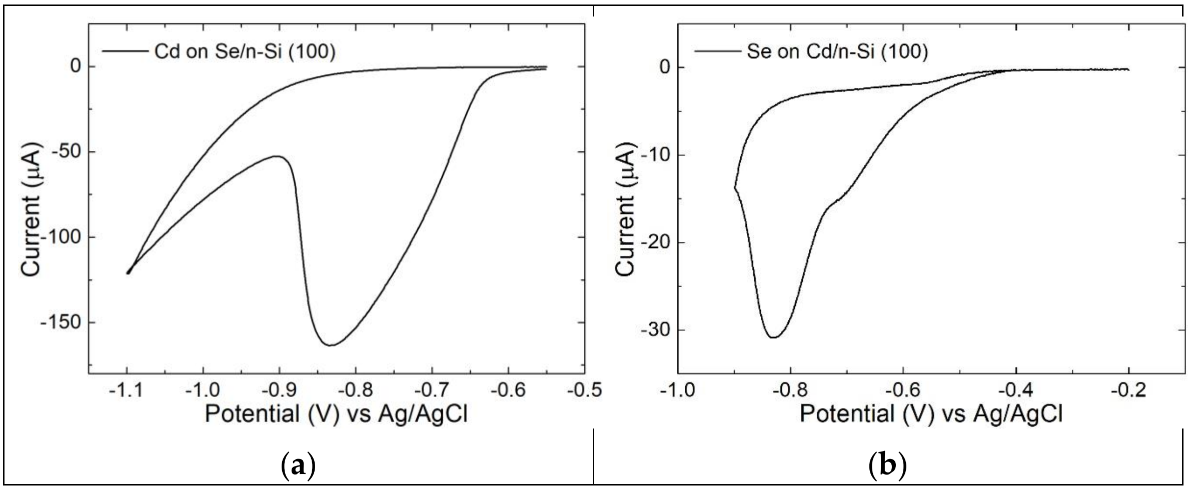

Figure S3. a) CV of Cd^{2+} solution on Se deposited on n-Si performed between -0.55 V and -0.9 V, scan rate 10 mV/s; b) a) CV of Se(IV) solution on Cd deposited on n-Si performed between -0.9 V and -0.2 V, scan rate 10 mV/s;.

© 2019 by the authors. Submitted for possible open access publication under the terms and conditions of the Creative Commons Attribution (CC BY) license (http://creativecommons.org/licenses/by/4.0/).
